# Pimavanserin in Parkinson’s Disease-induced Psychosis: A Literature Review

**DOI:** 10.7759/cureus.5257

**Published:** 2019-07-28

**Authors:** Rikinkumar S Patel, Jatminderpal Bhela, Muhammad Tahir, Sindhu Reddy Pisati, Sadaf Hossain

**Affiliations:** 1 Psychiatry, Griffin Memorial Hospital, Norman, USA; 2 Medicine, Windsor University School of Medicine, Monee, USA; 3 Internal Medicine, Penn Medicine Lancaster General Hospital, Lancaster, USA; 4 Neurology, Beth Israel Deaconess Medical Center, Boston, USA; 5 Psychiatry, Brookdale Hospital, Brooklyn, USA

**Keywords:** pimavanserin, parkinson’s disease, psychosis, treatment efficacy, drug safety

## Abstract

Pimavanserin was approved for treating Parkinson’s disease (PD) psychosis, based upon 21 completed studies. This review article is to understand PD psychosis and assess the efficacy and safety of pimavanserin. A literature search was carried out using the keyword “pimavanserin” and cross-referencing it with PD, psychosis, efficacy, safety and clinical trial. Participants in pimavanserin group were associated with a 5.79-point decrease in symptoms for PD psychosis (SAPS-PD) scale compared to the 2.73-point decrease seen in the placebo group (P < .001). There were statistically significant improvements in the persecutory delusions, ideas of reference, and global ratings of delusions in pimavanserin group. Pimavanserin was well tolerated with no significant adverse events or worsening of motor function. Pimavanserin at 34 mg daily was shown to be effective for PD-induced psychosis in past clinical trials.

## Introduction and background

Parkinson's disease (PD) is the most prevalent neurologic disorder that leads to increasing disability that is seen in about one percent of the elderly population (above 60 years) [1]. Per the National Institute of Neurological Disorders and Stroke, psychotic symptoms during the early stages of PD comprise of passage/presence hallucinations, visual hallucinations, and illusions. Whereas in the later stage of PD, auditory hallucinations are like non-comprehensible or non-verbal sounds that commonly occur several times in a day, lasting for few seconds to minutes in a quiet environment [2]. The prevalence of comorbid psychosis is 3.9% and the risk of worsening of disease severity is 1.4 times higher in the hospitalized PD patients [3].

A study was conducted in non-demented PD patients using fluorodeoxyglucose positron emission tomography (PET) to determine the characteristics of cerebral glucose metabolism in PD patients with visual hallucinations [4]. In patients with hallucinations, the relative regional cerebral glucose metabolic rate was higher in the frontal areas, especially in the left superior frontal gyrus. It was shown that relative frontal hyper-metabolism might be a feature of PD patients with visual hallucinations. When the hallucinating PD patients showed greater activation in the inferior frontal gyrus and the caudate nucleus, then the non-hallucinating PD patients showed significant activation in the parietal lobe and cingulate gyrus [4, 5].

Pimavanserin is Food and Drug Administration (FDA) approved in April 2016 for treating hallucinations and delusions in PD patients. The objective of this review is to understand the clinical stages of PD psychosis and assess the efficacy and safety of pimavanserin based upon the latest studies.

Literature searches on Medline and Embase were carried out (January 1, 2008 to December 1, 2018) using the keyword “pimavanserin” and cross-referencing it with Parkinson’s disease, psychosis, efficacy, safety, and clinical trial. Studies found through this search were further reviewed and screened to identify relevant studies.

## Review

Psychosis in the early stage of PD

According to a case series by Fenelon et al., 52 non-demented PD patients reported a feeling of presence (FP), i.e., the vivid sensation that somebody other than oneself is present nearby [6]. FP characteristics for the preceding month were recorded in a questionnaire survey. This group was compared with 78 PD patients without FP. Around half the patients said that they recognized the ‘identity’ of the presence. About 75% of patients said the FP was short-lasting, non-distressing, and occurred while indoors [6]. Most patients checked for a real presence, but their insight was preserved. In 31% of cases, the patients had unformed visual hallucinations with the FP [6].

Few past studies have found a relationship between the cognitive profile and the hallucination experienced in PD patients with formed hallucinations. Hallucinations in PD patients with unidentified content had more significant inhibitory function deficits than patients with acknowledged hallucinations [7]. Additional visual symptoms that may be observed include isolated diplopia, freezing (cessation when the patient is walking through narrow spaces), and spatial misjudgment [8,9].

Psychosis in the later stage of PD

With disease progression in PD patients, non-visual hallucinations including auditory, tactile, or olfactory types occur concurrently with visual hallucinations. These findings are not restricted to end-stage PD with dementia as it's also seen in patients with intact cognition levels and having a mini-mental state examination (MMSE) score of above 24 [10,11].

As per a study conducted in a clinical setting by Factor et al., about 16% of PD patients endorse delusions [12]. Unique delusions include the Capgras delusion (delusion about a familiar individual has been replaced by an imposter), reduplicative paramnesia (delusion about a place has been duplicated and is present at two locations at the same time) and the mirror sign, i.e., inability to recognize self in the mirror. The prevalence of delusion with severe cognitive decline is seen in dementia with Lewy bodies and Alzheimer’s disease [13]. A prospective study found that about 16.7% of PD patients with dementia endorse delusional symptoms [14].

Mechanism of action of pimavanserin

Pimavanserin is a partial inverse agonist and antagonist at serotonergic 5-HT2a receptors. The selectivity for 5-HT2a receptors by sparing the postsynaptic dopamine receptors differentiates it from other antipsychotics used for PD psychosis [15]. Pimavanserin has a higher affinity for 5-HT2a receptors, and a lower affinity to 5-HT2b, 5-HT2c, and dopaminergic (D3) receptors [16]. The active metabolite of pimavanserin, AC-279 has a half-life of 200 hours. Studies on animals have shown that there are reactive adaptations in serotonergic signaling, which includes upregulation of 5-HT2a mitochondrial RNA in the striatum [17]. Hence, pimavanserin specifically targets the 5-HT2a receptor and is highly effective.

Efficacy of pimavanserin

In a six-week, randomized, double-blinded, placebo-controlled (RDBPC) study, adults aged ≥ 40 years with PD psychosis were enrolled [18]. Participants were randomly assigned into groups and received pimavanserin 40 mg daily or placebo. The primary outcome measure in this trial was an antipsychotic effect that was assessed by independent raters using the PD-adapted scale for assessment of positive symptoms (SAPS-PD). Pimavanserin was associated with a 5.79-point decrease in SAPS-PD scale compared to the 2.73-point reduction in participants receiving matched placebo, that is a statistically significant difference of 3.06 (P < .001) [18].

In another RDBPC trial conducted over the eight weeks included patients at 1:1 ratio, and they received a placebo or pimavanserin [19]. Pimavanserin was started at 20 mg, and depending on the patient's clinical response, the dose was increased to 40 or 60 mg daily on day 8 and day 15, respectively. There was a statistically significant improvement in the global rating of hallucinations in the pimavanserin group (P = .02, effect size (ES) = .58). Also, some improvements in pimavanserin group was seen in SAPS delusion domain measures, including persecutory delusions (P = .009, ES = .41), and ideas of reference (P = .05, ES = .36). The participants in pimavanserin group showed improvement in the SAPS total domain score (P = .09, ES = .52), i.e., 40% improvement compared with an 11% improvement seen in the placebo group [19].

Safety of pimavanserin

The most common side effects comparing patients on pimavanserin relative to placebo groups were peripheral edema (7%) and confusion (6%) [20]. Other significant adverse drug events were increased risk of fall, urinary tract infections, and hallucinations (5%) and constipation (4%). It is safe to prescribe pimavanserin in patients on carbidopa/levodopa, as no significant drug interactions were observed [19]. It is recommended to reduce the pimavanserin dose by 50% when given along with cytochrome P450 (CYP450) inhibitor. On the contrary, pimavanserin dose may need to be increased if it is given with a CYP450 inducer. An insignificant QT interval prolongation for 9.6 milliseconds was seen in a RDBPC trial at a dose of 34 mg [19]. Pimavanserin is well-tolerated medication for managing psychosis in patients with PD with no serious adverse events [18].

## Conclusions

After reviewing existing literature, pimavanserin at a total daily dose of 34 mg (split into 17 mg two times a day) is useful for treating psychosis (hallucinations and delusions) in PD patients. Dose changes may be required when pimavanserin is used with CYP450 inhibitor or inducer. No dose changes are needed for patients with mild to moderate renal impairment, but pimavanserin is not recommended in PD patients with severe renal or hepatic impairment due to lack of evidence. Pimavanserin has a black box warning on its package labeling, as it increases mortality in older age patients with dementia-related psychosis, and clinicians need to be cautious with the use of pimavanserin with other drugs that prolong QT-interval. As pimavanserin is an effective and safe medication, it may replace current antipsychotic therapy for the treatment of psychosis associated with PD.

## References

[1] (2019). Parkinson disease guidelines. https://emedicine.medscape.com/article/1831191-guidelines.

[2] Ravina B, Marder K, Fernandez HH (2007). Diagnostic criteria for psychosis in Parkinson's disease: report of an NINDS, NIMH work group. Mov Disord.

[3] Imran S, Patel RS, Onyeaka HK (2019). Comorbid depression and psychosis in Parkinson’s disease: a report of 62,783 hospitalizations in the United States. Cureus.

[4] Nagano-Saito A, Washimi Y, Arahata Y (2004). Visual hallucination in Parkinson's disease with FDG PET. Mov Disord.

[5] Stebbins GT, Goetz CG, Carrillo MC, Bangen KJ, Turner DA, Glover GH, Gabrieli JD (2004). Altered cortical visual processing in PD with hallucinations: an fMRI study. Neurology.

[6] Fénelon G, Soulas T, Cleret de Langavant L, Trinkler I, Bachoud-Lévi AC (2011). Feeling of presence in Parkinson's disease. J Neurol Neurosurg Psychiatry.

[7] Boubert L, Barnes J (2015). Phenomenology of visual hallucinations and their relationship to cognitive profile in Parkinson’s disease patients: preliminary observations. SAGE Open.

[8] Nebe A, Ebersbach G (2007). Selective diplopia in Parkinson's disease: a special subtype of visual hallucination?. Mov Disord.

[9] Urwyler P, Nef T, Killen A (2014). Visual complaints and visual hallucinations in Parkinson's disease. Parkinsonism Relat Disord.

[10] Chou KL, Messing S, Oakes D, Feldman PD, Breier A, Friedman JH (2005). Drug-induced psychosis in Parkinson disease: phenomenology and correlations among psychosis rating instruments. Clin Neuropharmacol.

[11] Papapetropoulos S, Katzen H, Schrag A (2008). A questionnaire-based (UM-PDHQ) study of hallucinations in Parkinson's disease. BMC Neurol.

[12] Factor SA, Scullin MK, Sollinger A (2014). Cognitive correlates of hallucinations and delusions in Parkinson's disease. J Neurol Sci.

[13] Ballard C, Holmes C, McKeith I (1999). Psychiatric morbidity in dementia with Lewy bodies: a prospective clinical and neuropathological comparative study with Alzheimer's disease. Am J Psychiatry.

[14] Pagonabarraga J, Llebaria G, García-Sánchez C, Pascual-Sedano B, Gironell A, Kulisevsky J (2008). A prospective study of delusional misidentification syndromes in Parkinson's disease with dementia. Mov Disord.

[15] Meltzer HY, Mills R, Revell S, Williams H, Johnson A, Bahr D, Friedman JH (2010). Pimavanserin, a serotonin(2A) receptor inverse agonist, for the treatment of Parkinson's disease psychosis. Neuropsychopharmacology.

[16] (2019). Center for drug evaluation and research. https://www.accessdata.fda.gov/drugsatfda_docs/nda/2016/207318Orig1s000Approv.pdf.

[17] Zhang X, Andren PE, Svenningsson P (2007). Changes on 5-HT2 receptor mRNAs in striatum and subthalamic nucleus in Parkinson's disease model. Physiol Behav.

[18] Cummings J, Isaacson S, Mills R (2014). Pimavanserin for patients with Parkinson's disease psychosis: a randomised, placebo-controlled phase 3 trial. Lancet.

[19] Meltzer HY, Roth BL (2013). Lorcaserin and pimavanserin: emerging selectivity of serotonin receptor subtype-targeted drugs. J Clin Invest.

[20] Howland RH (2016). Pimavanserin: an inverse agonist antipsychotic drug. J Psychosoc Nurs Ment Health Serv.

